# Delayed Migration of Embolized Coil with Large Renal Stone Formation: A Rare Presentation

**DOI:** 10.1155/2014/687965

**Published:** 2014-12-24

**Authors:** Santosh Kumar, Kumar Jayant, Shrawan K. Singh, Kalpesh M. Parmar, Sudheer K. Devana, Gautam R. Choudhari, Ankur Mittal

**Affiliations:** Department of Urology, Postgraduate Institute of Medical Education and Research, Chandigarh 160012, India

## Abstract

Delayed bleeding following percutaneous nephrolithotomy (PCNL) usually occurs due to development of the pseudoaneurysm which can be successfully managed with coil embolization. However very few cases of such complications have been reported in the literature. Here we are reporting a case of delayed post-PCNL bleeding that occurred in a 53-year-old diabetic patient operated on for renal stone. Computed tomography scan revealed a presence of the pseudoaneurysm in the segmental branch of right renal artery, which was successfully managed with coiling. Patient remained asymptomatic for the next 9 years after which he again presented with similar complaints. X-ray KUB was done which revealed a 2.7 cms renal pelvic calculus with the migrated coil in its center and a left upper ureteric calculus. His routine haemogram, coagulogram, serum electrolytes, and liver function tests, renal function tests, vitamin D3, and PTH (parathyroid hormone) were within normal limits. He underwent left laparoscopic ureterolithotomy and right percutaneous nephrolithotomy (PCNL). Intraoperatively the migrated stainless steel embolization coil was seen engulfed all around by the multiple stones in the right renal pelvis. Postoperative period was uneventful. Later he was followed in the outpatient department and was doing well. To conclude, this is the only case report of development of a large calculus around a migrated embolization coil which was successfully managed with PCNL. PCNL offers better stone clearance in cases of stones being formed over foreign bodies like fragmented double J stents, fragmented nephrostomies, or migrated embolization coil.

## 1. Introduction

Kidney stones can be very painful and if left untreated may become bigger, leading to infection and impaired kidney function. Percutaneous means “through the skin” and nephrolithotomy means “taking stones out of the kidney.” Percutaneous extraction of the renal stone, that is, percutaneous nephrolithotomy (PCNL), was invented by Fernstrom and Johansson. They formed a percutaneous track for the specific purpose of removing an intrarenal stone. Since it has evolved it has been refined with time along with the development of various instruments, endoscopes, and other gadgets and has remained a standard treatment for all kinds of renal stones particularly large (>2 cms) or hard stones that fail to break with other treatment modalities such as shock wave lithotripsy [1, 2].

Postoperative complications are not unusual following anesthesia and surgery, and their incidence may be up to 10–20%. These include pulmonary complications as atelectasis, pneumothorax, hemothorax, or hydrothorax, pleural effusion, pulmonary edema, pneumonia, and acute respiratory distress syndrome. The other component of complications starts from the initial puncture with injury to surrounding organs (e.g., colon, spleen, liver, pleura, and lung). Other specific complications include postoperative bleeding and fever. Vascular injuries leading to renal hemorrhage are also a relatively common complication. Often this kind of bleeding is usually self-limited, mainly because of restrictive effect of the Gerota's fascia and retroperitoneum. Thus the actual incidence of hemorrhage requiring blood transfusion varies between 4% and 12% in the different studies. The vascular complications of this procedure include hematoma, pseudoaneurysm, and arteriovenous fistula, of which pseudoaneurysm is usually symptomatic only 24 hours after the procedure [3, 4].

Angiographic computed tomography (CT) scan has an important role in the diagnosis. The most common angiographic finding associated with delayed hemorrhage is the development of arterial pseudoaneurysm. Selective angiographic embolization is the treatment of choice for most patients with renal pseudoaneurysm [5]. Here we are reporting a case of post-PCNL bleeding due to development of the pseudoaneurysm which was successfully managed with coiling but later presented with the multiple large stones in renal pelvis secondary to migration of the coil.

## 2. Case Report

A 53-year-old diabetic man presented to our emergency department with history of the recurrent right loin pain in 2003. After evaluation he was diagnosed with right renal pelvis stone for which he underwent percutaneous nephrolithotomy (PCNL). The procedure was uneventful and the patient was discharged on the third postoperative day. A week after the PCNL, the patient returned to our emergency department with severe hematuria and clot retention. Routine blood investigations and coagulation parameters were within the reference ranges preoperatively. The patient was resuscitated with intravenous fluids and 3 units of packed red blood cells was transfused. His haemoglobin was 5 g/dL but coagulogram, serum electrolytes, and liver function tests and renal function tests were within normal limits. He had 2 additional episodes of severe hematuria with clots, despite conservative management. The urine was clear between the hematuria episodes. Ultrasound and computed tomography scans showed clots in the urinary tract without significant collection in the retroperitoneal space. The multiple episodes of gross hematuria which occurred during the period of conservative management changed the hemodynamic parameters, and the hemoglobin level decreased to 4 mg/dL; the patient underwent angiography which showed a pseudoaneurysm arising from segmental branch of right renal artery, although there was no active contrast leak. Later angioembolization was done with a metallic steel coil in 2003. Following the procedure his hematuria was settled and he was discharged in the satisfactory condition.

**Figure 1:**
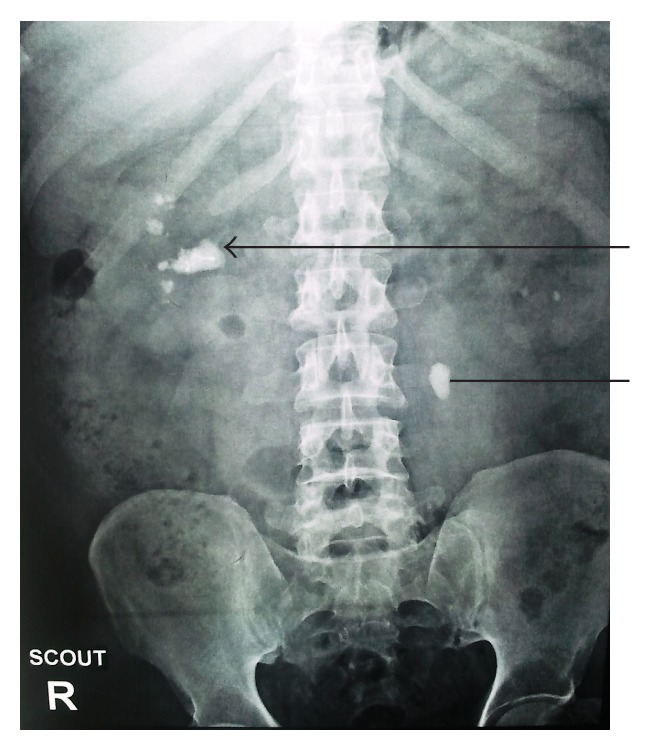
Plain X-ray KUB showing right renal pelvic calculus with the stainless steel embolization coil within it and multiple calyceal stones (arrow). Left upper ureteric stone was also seen (straight line).

He remained asymptomatic till 2011 when he started developing bilateral dull aching loin pain. He presented to our outpatient department in 2012 with the increasing loin pain more on right side than left. He was evaluated and X-ray KUB was done which revealed a 2.7 cms renal pelvic calculus with the embolization coil in its center with multiple calyceal stones in the right pelvis and a left upper ureteric calculus (Figure 1). His routine haemogram, coagulogram, serum electrolytes, and liver function tests were within normal limits. His serum creatinine and urea were 1.0 mg/dL and 27 mg/dL. Vitamin D3 and PTH (parathyroid hormone) were within normal limit, that is, 52 nmol/L and 21 pgm/mL, respectively, and urine culture was sterile. His random blood sugar was persistently high in the range of 240–290 mg/dL, so he was switched from oral metformin to injectable regular insulin on sliding scale in view of scheduled surgical candidate.

**Figure 2:**
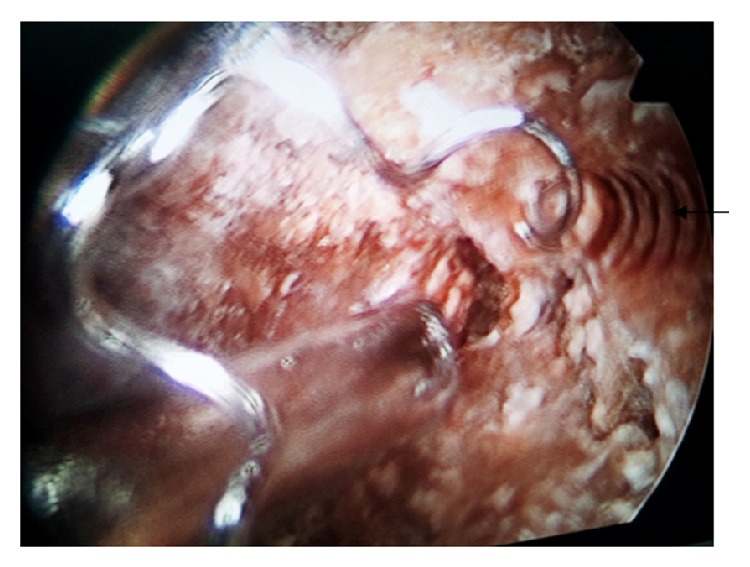
Intraoperative picture showing the renal pelvic stone with the migrated embolization coil (arrow). 6Fr ureteric catheter and the Amplatz sheath with nephroscope is also seen.

**Figure 3:**
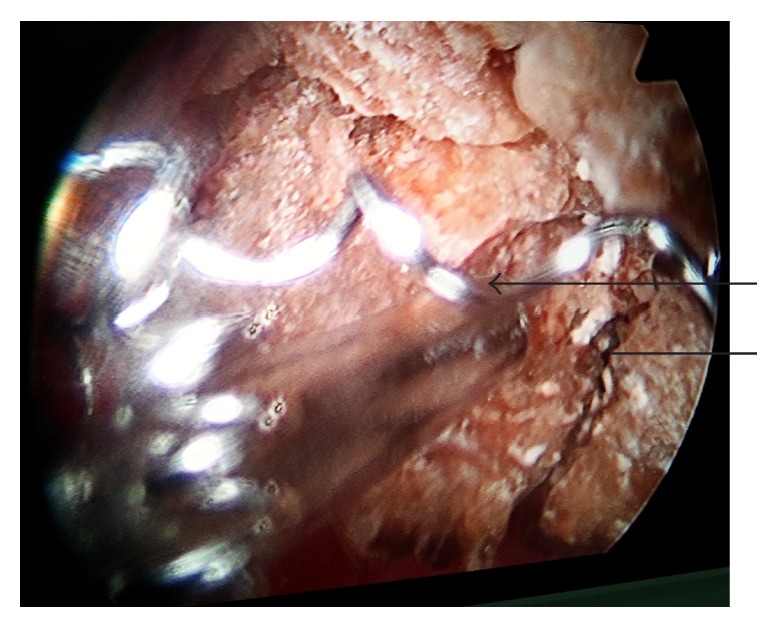
Intraoperative picture showing the renal pelvic stone (straight line) with the migrated stainless steel embolization coil (arrow) as nidus in the pelvicalyceal system.

**Figure 4:**
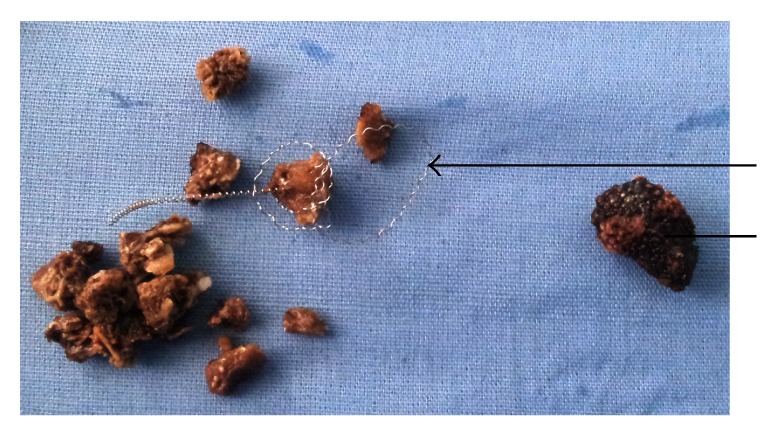
The fragmented renal pelvic stone (straight line) with the migrated embolization coil (arrow head).

He was taken for surgery and he underwent left laparoscopic ureterolithotomy and right percutaneous nephrolithotomy (PCNL). Intraoperatively the migrated stainless steel embolization coil was seen being engulfed all around by the multiple stones in right pelvis (Figures 2 and 3). We used pneumatic lithotripsy and complete retrieval of the all pelvic calculi along with the migrated embolization coil was done (Figure 4). Postoperative period was uneventful. He did not have any significant bleeding and his blood sugar was maintained within normal range. Nephrostomy tube was removed on the postoperative day 4 and following which he was discharged on the postoperative day 5 on injectable insulin glargine and insulin lispro. Later he was followed in outpatient department and was doing well.

## 3. Discussion

Percutaneous nephrolithotomy (PCNL) provides urologists with a safe and effective way to remove kidney stones using a minimally invasive technique thus considered as the treatment of choice for the management of renal stones larger than 2 cms in size. Post-PCNL bleed is a known complication with an incidence of 2–20%, while renal pseudoaneurysm is rare but clinically very significant, because of delayed bleeding following PCNL. As per recent literature search, the reported incidence of renal pseudoaneurysm following PCNL is low (0.7%–1.2%). Although a high index of clinical suspicion is of the utmost importance, CT angiography is considered as the best modality required to identify the source of bleeding. Therapeutic angiography, usually by superselective embolization of the injured vessel, has evolved as the treatment of choice with success rates that exceed 90% and has largely replaced the need for emergency surgery [6]. Stainless steel or titanium coils are routinely being used for angioembolization of renal pseudoaneurysms or arteriovenous fistulas [7]. Our index patient in this report also had recurrent post-PCNL bleeding secondary to development of pseudoaneurysm involving the segmental branch of right renal artery which was successfully managed with the angioembolization using steel coil.

Complications can occur following angioembolization in 10% of cases like postembolization syndrome, renal insufficiency, embolization of nontarget organs, infective complications, and migration of embolization coils. The coil migration most commonly occurs during the time of the procedure or in the immediate postprocedure period. Delayed coil migration is a very rare phenomenon. After extensive search of literature we found very few cases of the delayed migration of embolization coils following angioembolization [8]. Our patient in this report presented with features of the renal stones secondary to coil migration, approximately 9 years after angioembolization.

The possible mechanism behind such erosion and migration of coil into the PCS is that during PCNL there might have been calyceal tears at some places which later on got healed. Such healed calyceal areas are inherently weak and may get eroded by metallic coils slowly over a period of time due to inflammation leading to migration of these coils into the PCS. With time these migrated coils can get encrusted and act as nidus for the stone formation leading to obstruction and UTI causing flank pain, fever, and hematuria. Rutchik and Wong in 2002 showed a migrated embolization coil into the PCS with stone formation in a 36-year-old woman who presented to emergency with gross hematuria which was managed with ureteroscopy [9]. Savoie et al. and Reed et al. in 2007 reported two cases of migrated embolization coils being passed in urine by the patients [10, 11]. In the previously published case reports of migrated coils patients presented to emergency with hematuria but our patient presented with lumbar pain and he did not have any episode of hematuria following angioembolization.

This is the only case report till now which presented the development of multiple calculi around a migrated steel embolization coil which was then managed with PCNL. PCNL offers better stone clearance in the cases of stones being formed over foreign bodies like fragmented double J stents, fragmented nephrostomies, or migrated embolization coil [12]. Our case is interesting in the sense that a complication of PCNL, that is, delayed bleeding secondary to segmental renal artery pseudoaneurysm, was managed by angioembolization and subsequently right sided renal stone developed due to delayed migration of metallic angioembolization coil, which in turn was successfully managed by PCNL.

## 4. Conclusion

On the basis of this clinical experience, our take-home messages are the following.

- Delayed migration of coils can occur following angioembolization for PCNL bleeding.
- This migrated embolization coil acts as a nidus for stone formation.
- The percutaneous nephrolithotomy (PCNL) still remains the treatment of choice for removal of such stones.
